# The Screening Performance of Serum 1,3-Beta-D-Glucan in Patients with Invasive Fungal Diseases: A Meta-Analysis of Prospective Cohort Studies

**DOI:** 10.1371/journal.pone.0131602

**Published:** 2015-07-06

**Authors:** Tie-Ying Hou, Shou-Hong Wang, Sui-Xin Liang, Wen-Xin Jiang, Dan-Dong Luo, De-Hong Huang

**Affiliations:** 1 Medical Department of HAI Control, Guangdong General Hospital, Guangdong Academy of Medical Sciences, Guangzhou, Guangdong province, 510080, China; 2 Intensive Care Unit, Guangdong General Hospital, Guangdong Academy of Medical Sciences, Guangzhou, Guangdong province, 510080, China; 3 Intensive Care Unit, Guangdong General Hospital, Guangdong Academy of Medical Sciences, Guangzhou, Guangdong province, 510080, China; 4 Neonatal Intensive Care Unit, Guangdong General Hospital, Guangdong Academy of Medical Sciences, Guangzhou, Guangdong province, 510080, China; 5 Cardiac Surgery intensive care unit, Guangdong General Hospital, Guangdong Academy of Medical Sciences, Guangzhou, Guangdong province, 510080, China; 6 Guangzhou Hospital of TCM, Guangzhou, Guangdong province, 510130, China; The University of Texas at San Antonio, UNITED STATES

## Abstract

The serum 1,3-beta-D-glucan (BG) assay aids in the early diagnosis of invasive fungal diseases (IFDs) and has been approved for their diagnosis. However, reports on the screening performance of BG are scarce. We performed a meta-analysis of data extracted from only prospective cohort studies to evaluate the screening performance of the BG assay in the diagnosis of IFDs. We specifically searched 4 databases (the PubMed, Web of Science, Elsevier, and Cochrane Collaboration databases) according to EORTC-MSG criteria. A total of 1068 patients in 11 studies were analyzed. Deeks’ funnel plot asymmetry test suggested a low likelihood of publication bias for the included studies (p = 0.055). The pooled sensitivity, specificity, positive likelihood ratio, negative likelihood ratio, diagnostic odds ratio, and area under the summary receiver operating characteristic curve, with 95% confidence intervals, were 0.75(0.63,0.84), 0.87(0.81,0.92), 5.85(3.96,8.63), 0.30(0.20,0.45), 19.53(11.16,34.18), and 0.89(0.86,0.91), respectively. The findings of this meta-analysis suggest that the BG assay is a useful screening tool with high sensitivity and specificity for discriminating between patients with and without IFDs. In clinical practice, BG assay results should be evaluated together with clinical and microbiological findings.

## Introduction

Invasive fungal diseases (IFDs) are serious complications in patients with disease-related or iatrogenic immunosuppression [1,2] and in patients who are dependent on various types of supportive care [3–5]. In recent years, the incidence of IFDs has been increasing [6,7]. In addition, IFDs are associated with considerable morbidity, including mortality rates of 30–70% due to aspergillosis and of 40–50% due to candidiasis [8,9]. Early administration of antifungal therapy is important [10]. However, the diagnosis of IFDs is challenging because the clinical signs and conventional microbiological and histological techniques are generally not sensitive [11–13].

The serum 1,3-beta-D-glucan (BG) assay, derived from the major cell wall component of various medically important fungi, has been developed and frequently used [14–16]. The results of this assay have been included in the revised diagnostic criteria for IFDs of the European Organization for the Research and Treatment of Cancer/Mycoses Study Group (EORTC/MSG) [17]. However, the test performance varies. Systematic reviews have been conducted to investigate the diagnostic accuracy of the BG assay in the diagnosis of IFDs in patients with Pneumocystis jiroveci pneumonia, invasive candidiasis, and/or invasive aspergillosis [18–20]. In the present study, we performed a meta-analysis of data extracted from only prospective cohort studies to focus on the performance of the BG assay in screening IFDs.

## Materials and Methods

### Study selection and identification

We searched four databases (the PubMed, Web of Science, Elsevier, and Cochrane Collaboration databases) for records of studies that evaluated the diagnostic performance of serum BG for IFDs from January 2004 through April 2014. The search key words used were as follows: glucan, fungal disease, fungal infection, beta glucan, mycoses, candidiasis, candidemia, aspergillosis, and Aspergillus. The syntax was as follows: “glucan” OR “beta(β) glucan” AND “fungal infection”, “fungal disease”, “mycoses”, “Aspergillus”, “aspergillosis”, “candidiasis”, OR “candidemia”. Using the above search strategy, abstracts were identified and screened by 3 authors (TY.H., SH.W., and SX.L.) without language restrictions. Potentially relevant studies with full text were included based on the following inclusion criteria: (1) the EORTC/MSG criteria were treated as the reference standard for the classification of IFDs as proven, probable, or possible, independent of the BG test results[17,21]; (2) the data extracted as true-positive, false-positive, true-negative, or false-negative results of the BG test were reported independently or could be calculated using the data provided in the manuscript; (3) BG measurements were performed in a homogenous cohort of patients who were at risk for IFDs (data for healthy individuals used as controls were excluded from the analysis to avoid overestimating the specificity of BG testing); and (4) cutoffs of 80 pg/ml for the Fungitell assay, 11 pg/ml for the Wako BG assay, 20 pg/ml for the Fungitec G test, and 10 pg/ml for the GKT-25M were used because these cutoffs are considered to be equivalent [18,19]. Studies including fewer than 10 patients were excluded from the analysis to avoid selection bias.

Two reviewers (WX.J. and DD.L.) judged the study eligibility while screening the citations. The above criteria had to be agreed upon by 3 authors (TY.H., WX.J., and DH.H.) for inclusion in the analysis.

The quality of the included studies was assessed by 3 authors (TY.H., SH.W. and DH.H.) using the Quality Assessment of Diagnostic Accuracy Studies (QUADAS) tool, which is based on 14 items that were developed to assess the quality of studies investigating diagnostic tests[22,23]. Each item was scored as “Yes”, “No” or “Unclear”, and the agreement of the 3 authors was required.

### Data Analysis

To evaluate the performance of the BG test in screening for IFDs, patients with demonstrated or probable IFDs who were diagnosed according to the EORTC/MSG classification were compared with control patients, and patients with possible IFDs were also included in the analysis. Because we focused on screening performance, a positive test result was defined as 1 positive BG value based on the BG cutoff level used in each study. The pooled sensitivity and specificity, positive likelihood ratio (PLR), negative likelihood ratio (NLR), diagnostic odds ratio (DOR), and area under the summary receiver operating characteristic curve (AUC), with 95% confidence intervals (95% CIs), were calculated using a random-effects model [24]. A test for inconsistency (I^2^) was used to assess heterogeneity [25]. The possibility of publication bias was explored using Deeks’ funnel plot asymmetry test plots for DORs [26]. The presence of a threshold effect on the performance of the BG assay was evaluated using Spearman’s correlation coefficient between the logits of sensitivity and specificity [27]. The Midas module available in Stata software (version 11, Stata Corporation., http://www.stata.com) was used for the analysis. All of the statistical tests were two sided, and P values of 0.05 were considered statistically significant.

## Results

### Characteristics of the included studies

**Fig 1:**
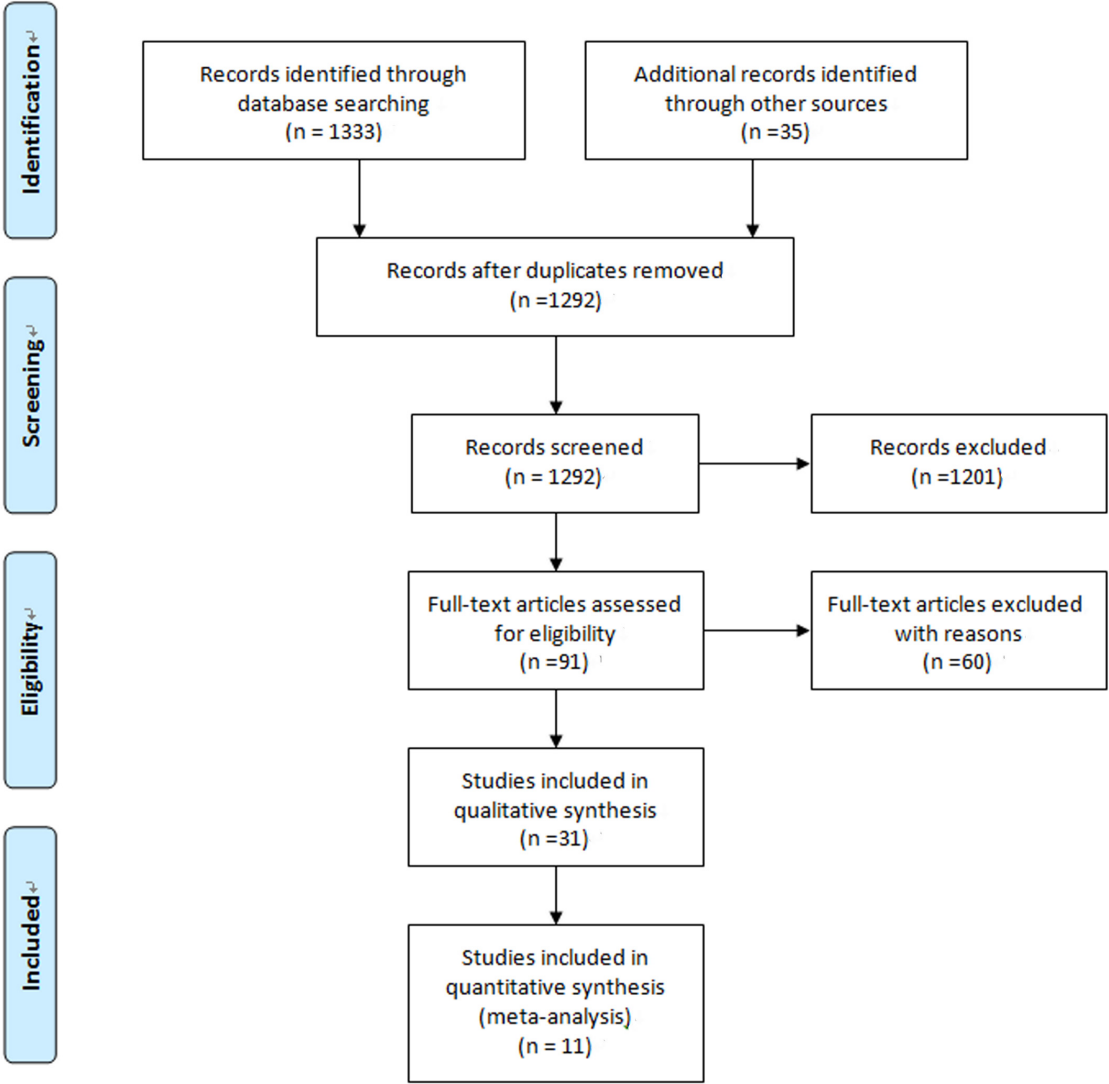
Flow chart showing the study selection process.

There were 1292 potentially relevant articles, of which 91 full-length articles were selected for a detailed analysis based on their title or abstract. The selection process for study inclusion is shown in Fig 1, and 11 prospective cohort studies met the inclusion criteria [6,7,9,10,12,28–34]. The characteristics of the 11 studies are summarized in Table 1. Among the 11 studies, 5 included patients with hematological malignancy (HM) or other serious tumors [6,12,28,29], 1 included patients who were liver transplant recipients[30], and the remaining 5 studies assessed hospitalized patients who might be at high risk for IFDs. The characteristics of the control groups varied. A screening strategy was used to measure the BG levels in blood samples in 9 studies. The 11 studies included 5 assays: 6 used Fungitell (cutoff value: 80 or 120 pg/ml), 2 used the Fungitec G test (cutoff value: 20 pg/ml), 2 used the Wako BG test (cutoff value: 7 or 11 pg/ml), and 1 used the GKT-25M set (cutoff value: 10 pg/ml). All of the studies, which comprised 1068 patients, employed the EORTC/MSG criteria as the reference standard for IFDs, as shown in Table 2.

**Table 1:** Characteristics of prospective cohort studies including BD Testing for the Diagnosis of IFDs.

Author/year	Population	Frequency of BG screening	BG assay	Cutoff (pg/ml)	Total No. of patients	proven or probable cases	possible cases	IA	IC
Kawazu/2004	patients with HM	Once per week	Wako	11	96(149 episodes)	11	13	11	0
Horiguchi/2004	patients with HM	Several samples were available for some patients	Fungitec G	20	58(69 episodes)	8		8	0
Pazos/2005	patients with HM	Twice per week	Fungitell	80	37	8	3	8	0
Akamatsu/2007	living donor liver transplant recipients	once per week for 3 months and once per month for 1 year	Fungitec G	20	180	24	unclear	5	14
Senn/2008	patients with HM	Twice per week	Wako	11	95(173 episodes)	32	30	13	15
Hachem/2009	patients with HM and other tumors	Twice in week 1 and once per week for 12 weeks	Fungitell	80	78	62	unclear	22	23
Zhao/2009	patients with hematologic or other malignant disorders	Twice per week	GKT-25M	10	130	22	7	4	2
Acosta/2011	patients with various diseases	Twice per week	Fungitell	80	51	13	unclear	10	0
Posteraro/2011	patients with various diseases	once per week	Fungitell	80	95	14	unclear	14	1
Mohr/2011	patients with various diseases	Twice per week	Fungitell	80	57	9	6	0	15
Bono/2011	patients with various diseases	Single sample per patient	Fungitell	80	152	53	47	53	0

IA,invasive aspergillosis;IC,invasive candidiasis

**Table 2:** Screening performance of invasive fungal infection based on data from different prospective studies.

Author/year	Reference standard	True positive	False negative	False positive	True negative
Kawazu/2004	EORTC/MSG	6	7	2	134
Horiguchi/2004	EORTC/MSG	7	1	9	52
Pazos/2005	EORTC/MSG	7	1	3	26
Akamatsu/2007	EORTC/MSG	14	10	26	130
Senn/2008	EORTC/MSG	30	30	12	101
Hachem/2009	EORTC/MSG	37	21	2	18
Zhao/2009	EORTC/MSG	18	4	19	89
Acosta/2011	EORTC/MSG	11	2	7	31
Posteraro/2011	EORTC/MSG	15	1	5	74
Mohr/2011	EORTC/MSG	14	1	14	28
Bono/2011	EORTC/MSG	79	21	9	43

### Quality assessment of the included studies

**Fig 2:**
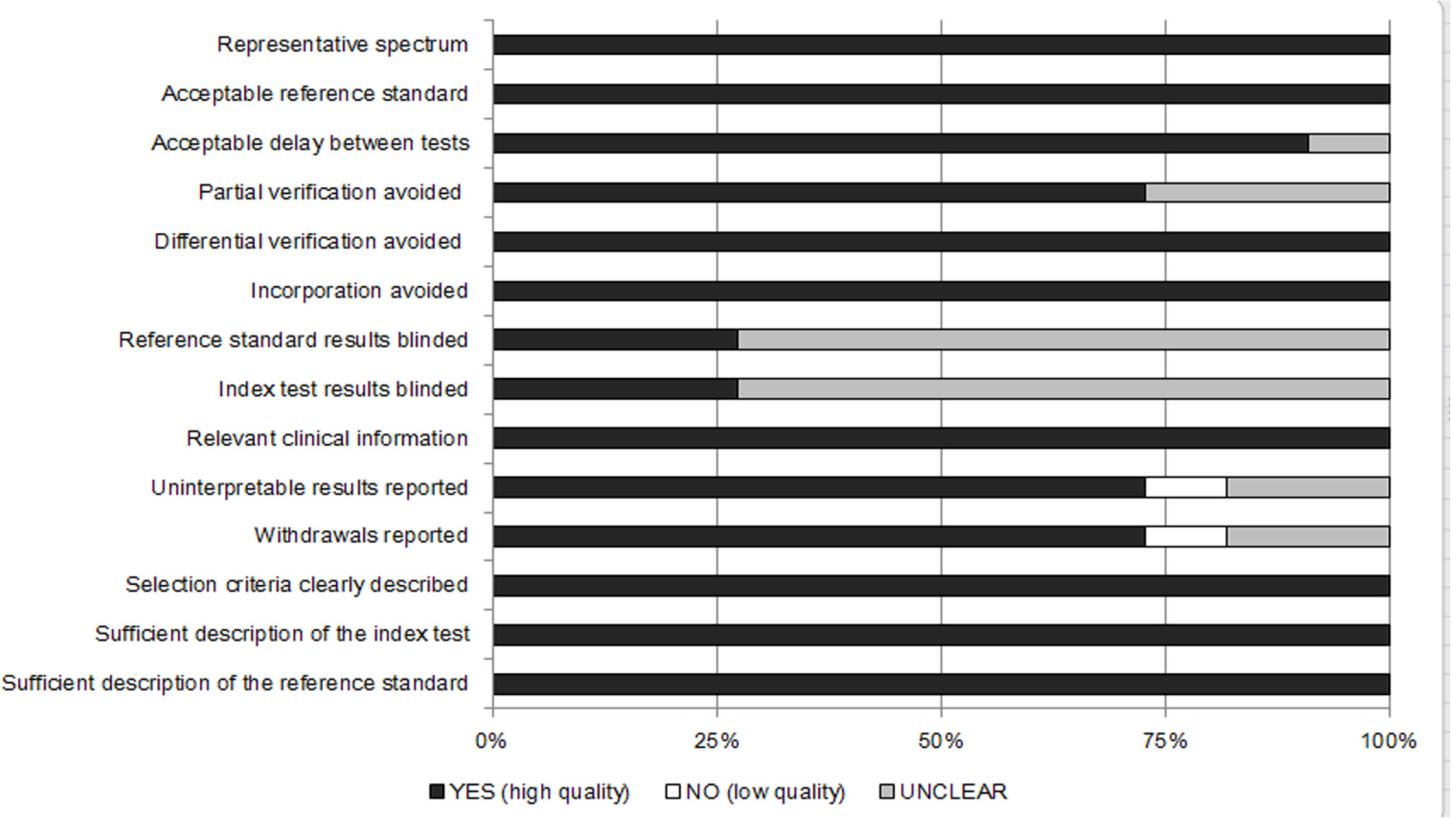
Summary of the methodological quality assessment of the reviewed studies according to the 14 QUADAS criteria.

Fig 2 summarizes the findings of the cumulative methodological quality assessment of all 11 studies using the QUADAS tool. All of the studies employed a representative spectrum and an acceptable reference standard and avoided differential verification and incorporation bias. The majority of the studies did not report whether the reference standard results or the index test results were blinded.

### Performance in screening for IFDs

**Fig 3:**
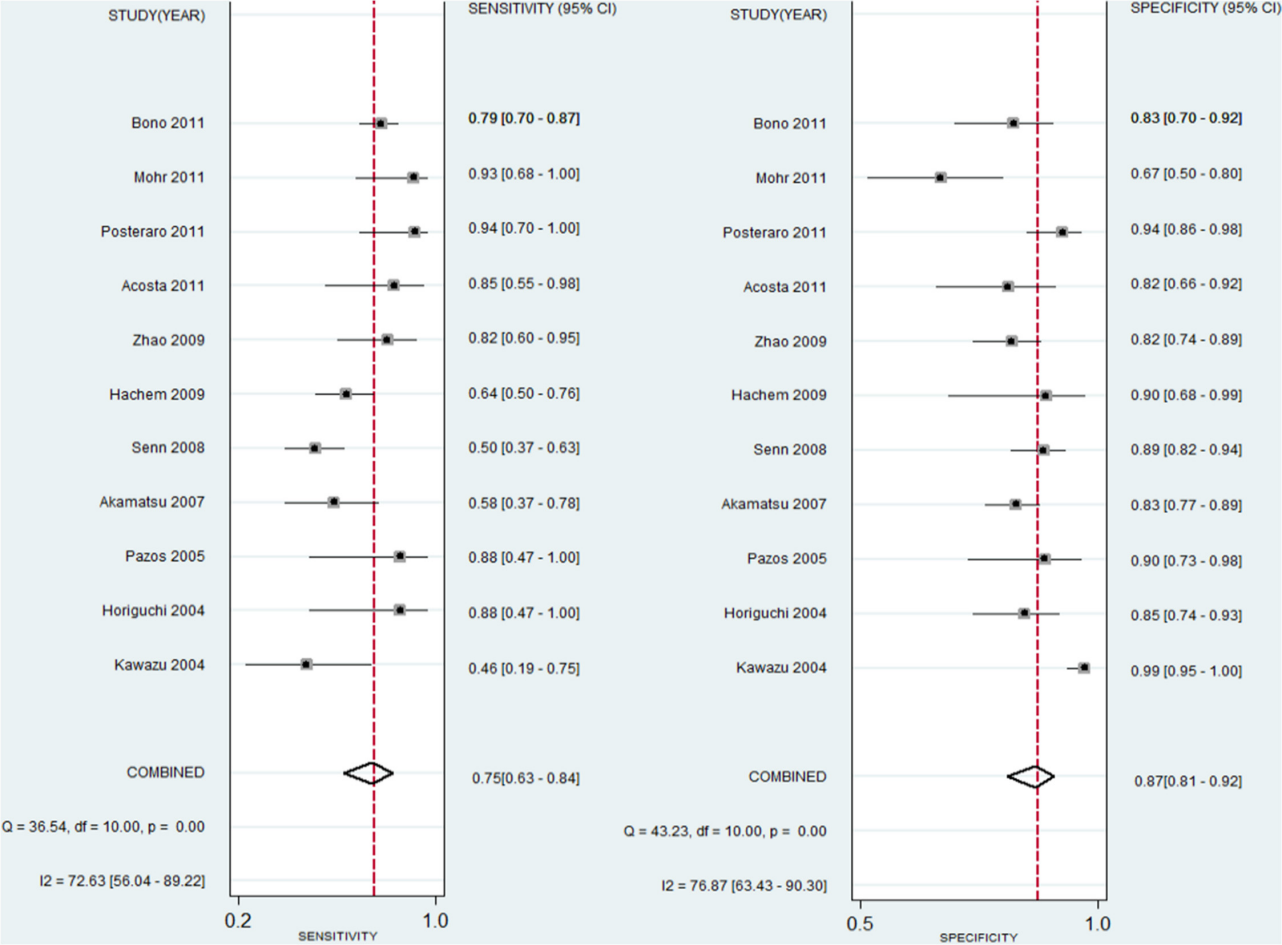
Forest plot of the pooled sensitivity and specificity of the BG assays for the diagnosis of IFDs.

**Fig 4:**
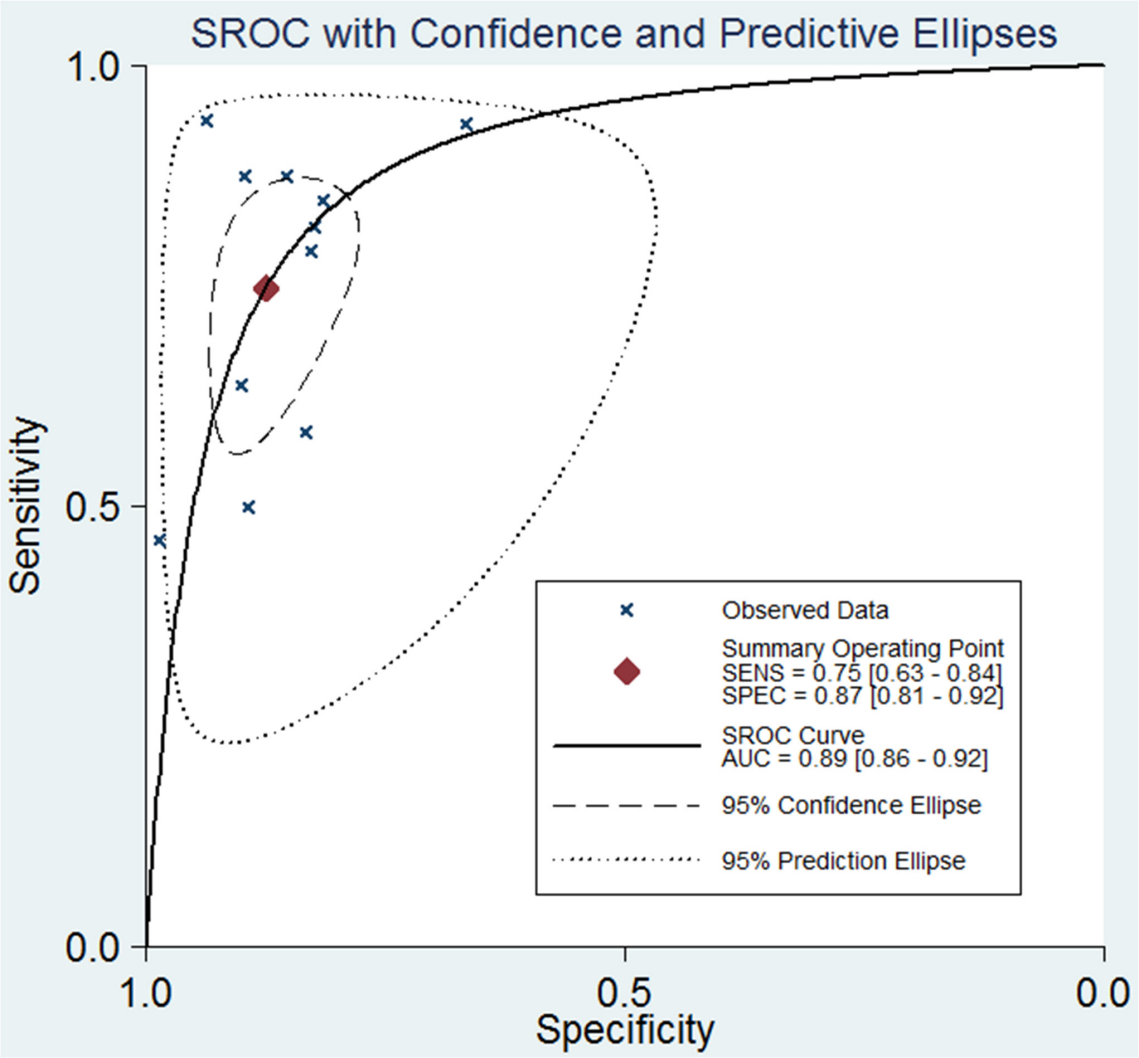
Summary receiver operating characteristic curve plots for the sensitivity and specificity for the diagnosis of IFDs.

**Fig 5:**
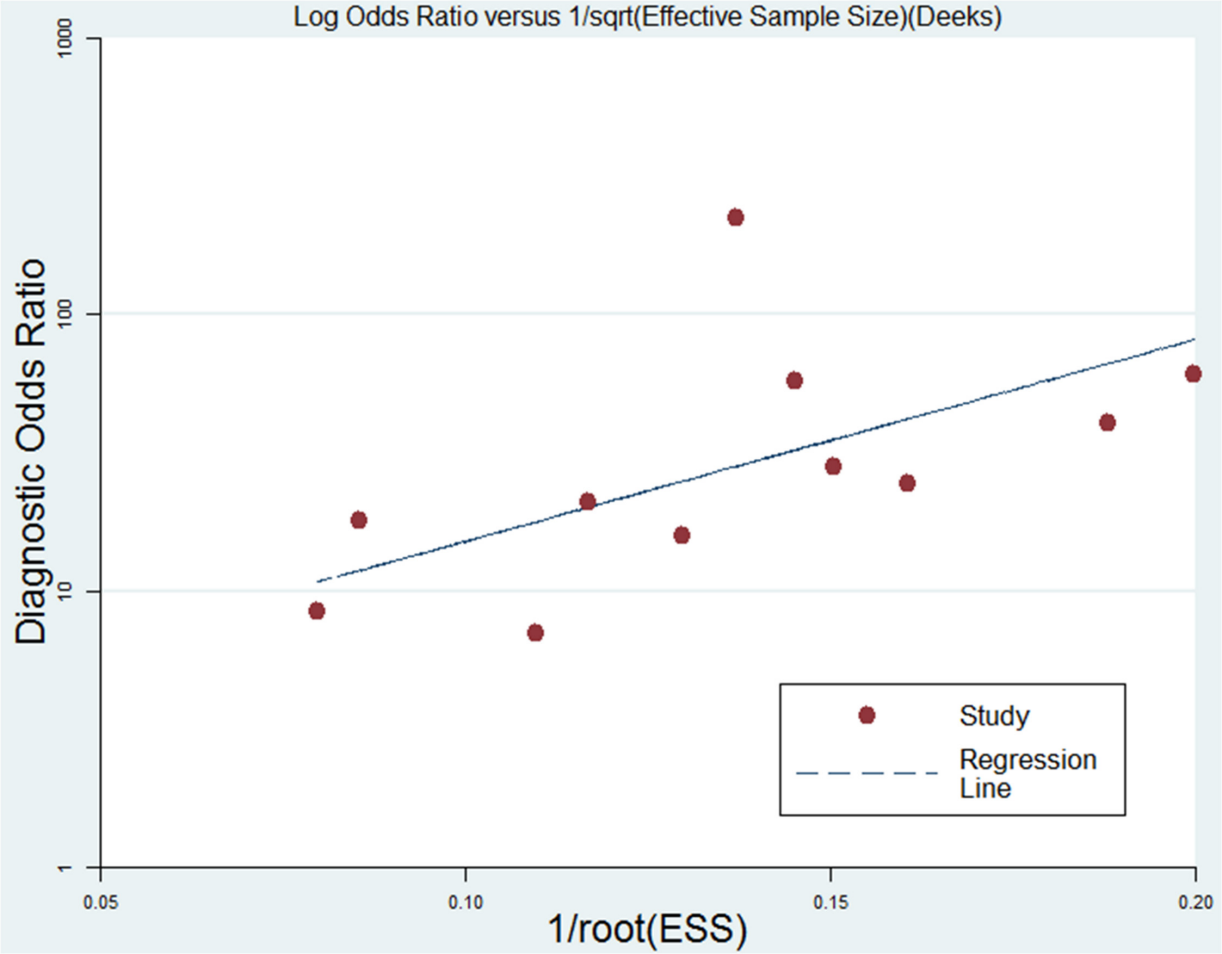
Linear regression of Deeks’ funnel plot asymmetry test for DORs (P = 0.055).

The pooled sensitivity, specificity, PLR, DOR, and AUC, with 95% CIs, are summarized in Table 3 and Fig 3. A similar subgroup analysis was performed (see Table 3). Relatively high inter-study heterogeneity was noted in 11 studies, and the I^2^ index was74.35% (95% CI: 59.87,98.33%). The SROC curves are displayed in Fig 4. The AUC value was 0.89 (95% CI: 0.86–0.91). Publication bias was evaluated using Deeks’ funnel plot asymmetry test, as shown in Fig 5.

**Table 3:** Pooled Test Performance of the Studies Included in the Meta-Analysis.

Test		No.of reports	Pooled SE(95%CI)	Pooled SPE(95%CI)	Pooled PLR(95%CI)	Pooled NLR(95%CI)	Diagnostic Odds Ratio	AUC(95%CI)
Kind of mycosis	Candidiasis	4	0.80(0.67,0.90)	0.77(0.67,0.89)	3.58(1.22,6.87)	0.26(0.10,0.57)	25.43(13.101,49.86)	0.88(0.83,0.98)
	Aspergillosis	6	0.73(0.62,0.86)	0.81(0.64,0.85)	5.57(3.89,6.23)	0.34(0.12,0.48)	23.15(10.4,58.90)	0.85(0.70,0.95)
Assay type	the Fungitell assay only	6	0.82(0.68,0.90)	0.86(0.77,0.92)	5.69(3.46,9.35)	0.22(0.12,0.39)	26.52(11.72,60.07)	0.90(0.88,0.93)
Pooled studies^a^		11	0.75(0.63,0.84)	0.87(0.81,0.92)	5.85(3.96,8.63)	0.30(0.20,0.45)	19.53(11.16,34.18)	0.89(0.86,0.91)

NLR, negative likelihood ratio; PLR, positive likelihood ratio; DOR, Diagnostic Odds Ratio; AUC, the area under the summary receiver operating characteristic curve;^a^:I^2^ = 74.35%,95% CI:59.87–98.33%

## Discussion

In this meta-analysis, the I^2^ of the 11 studies was 74.35% (95% CI: 59.87,98.33%), representing moderate heterogeneity [25,26,35]. In addition, a weak negative correlation was observed between the logits of sensitivity and specificity calculated for each of the 11 studies (Spearman’s correlation coefficient = -0.11), indicating a minor effect of the diagnostic threshold (cutoff level) on the performance of the BG measurement[19]. Regarding Deeks’ funnel plot asymmetry test, as shown in Fig 5, the non-significant slope coefficient (p = 0.055) suggested relative symmetry of the data and a low likelihood of publication bias.

Five types of BG assays, with different cutoff values, were included in the analysis. The different cutoffs for BG levels used may have altered the true-positive results and potentially created a bias toward the assessment of certain patients with false-positive results. In the study inclusion process, if a study reported BG data with different cutoff values, we included data referring to cutoffs of 80 pg/ml for the Fungitell assay, 11 pg/ml for the Wako BG assay, 20 pg/ml for the Fungitec G test, and 10 pg/ml for the GKT-25M. All of these cutoffs were considered to be equivalent, as indicated in several studies [18,19].

This meta-analysis included data for 1068 patients extracted from 11 prospective cohort studies with strict criteria for patient enrollment. In particular, data for healthy individuals used as controls were excluded from the analysis to avoid overestimating the specificity of the BG testing[36]. Therefore, all of the selected patients and controls at high risk for IFDs can be considered as highly representative of the actual clinical setting [9].

When evaluating the various patient categories, we included patients with possible IFDs because they represent a large number of patients in clinical practice. In addition, a certain proportion of possible IFD patients might have a true IFD, and therefore, the exclusion of data on possible IFD cases may lead to overestimating the assay sensitivity[37,38].

In comparison with certain meta-analyses evaluating the diagnostic accuracy of BG assays, including cohort studies and case-control studies, the sensitivity and specificity of the BG measurements in our meta-analysis appeared to be lower (0.75 vs. 0.78) and higher (0.87 vs. 0.80), respectively[19]. The use of case-control studies may bias results[39]. Lamoth et al. conducted a meta-analysis of only 6 cohort studies investigating the performance of BG assays in the diagnosis of IFD in hemato-oncological patients, reporting a lower sensitivity (0.70) and a higher specificity (0.91)[36]. However, the findings of the earlier meta-analysis should be interpreted with the high heterogeneity, ranging from 79% to 96%, in mind.

In general, an AUC value between 0.80 and 0.90 is considered to indicate good screening performance [18,40–41]. Although the AUC values in the 11 prospective cohort studies varied, the present meta-analysis demonstrated that serum BG measurement had good performance in screening for IFDs, with an AUC value of 0.89.

This meta-analysis has certain limitations. First, the quality assessment using the QUADAS tool showed that in the majority of the studies, information on blinded reference standard results and index test results was not reported. Second, there were only 4 studies on the analysis of invasive candidiasis, so the pooled screening performance-related values for invasive candidiasis (such as pooled sensitivity) should be considered carefully. Third, the performance of the BG assay in screening for the Pneumocystis jiroveci pneumonia was not assessed in this study due to a lack of data.

In conclusion, the findings of this meta-analysis suggest that the BG assay is a useful screening tool with high sensitivity and specificity for discriminating between patients with and without IFDs. In clinical practice, BG assay results should be evaluated together with clinical and microbiological findings. Certain issues regarding the optimal utilization of BG testing require further evaluation, especially the optimal sampling strategy for patients who are at high risk [20].

## Supporting Information

S1 Checklist PRISMA 2009 checklist in this meta-analysis. (DOC)
